# P262: Successful implementation of an infection control programme in a tertiary care oncology centre from a developing country

**DOI:** 10.1186/2047-2994-2-S1-P262

**Published:** 2013-06-20

**Authors:** A Ghafur, P Vidyalakshmi, K Chandra

**Affiliations:** 1 Apollo Speciality Hospitals, Chennai, India

## Introduction

Infection Control principles and practices have rarely been given importance in developing countries.

## Objectives

Against the rising antimicrobial resistance and realising the importance of preventive measures, we would like to highlight our achievement in establishing an infection control setup in our hospital, a tertiary care oncology and neurosurgical centre.

## Methods

1) Monitoring, surveillance and education on infection control practices is a dynamic process in our institution. 2) Isolation precautions are strictly followed for patients with Multidrug Resistance bacteria. 3) Hand hygiene compliance is monitored regularly and feedback given. Regular educational sessions are conducted. 4) Introduced tight monitoring of higher end antibiotics in 2007, with stricter implementation since 2008. A list of restricted antibiotics was prepared and discussed with the other doctors. 5) When a high end antibiotic is used, second opinion from an ID physician was made mandatory. If there is any violation then the infection control team and management steps in.

## Results

Very good compliance (>80%) to high end antibiotic policy, which is well documented and tracked since 2008 recorded as hard and soft copies. Compliance to surgical prophylaxis policy stands at 80%. Hospital accreditation board inspectors rate our infection control and antibiotic stewardship practices highly (documented). We document minutes of infection control meetings, explaining the step by step implementation of infection control .Our achievements are published in both Indian (Journal of Association of physicians of India) and International medical journals (Antimicrobial resistance and Infection control) and have been presented in various conferences (ICAAC,ICHS,CIDSCON,ISAAR,HISICON etc.). Our department consultant spearheaded “The Chennai declaration” which was historic event which brought together the medical societies in India. Our statements are backed by documentary evidences.

## Conclusion

Our success shows that infection control and antimicrobial stewardship can be successfully implemented in hospitals in developing countries. Our department has contributed to the success of infection control of not only our hospital but to the need of the country as well.

## Competing interests

None declared.

